# Assessing likelihood of using the Bruininks–Oseretsky test of motor proficiency to predict preclinical performance of dental students

**DOI:** 10.1002/cre2.217

**Published:** 2019-07-07

**Authors:** Ammar Musawi, Travis Barrett, Hamid Nurrohman, Shalini Bhatia, Kneka Smith

**Affiliations:** ^1^ Missouri School of Dentistry and Oral Health A.T. Still University Kirksville Missouri; ^2^ Department of Research Support A.T. Still University Kirksville Missouri

**Keywords:** Bruininks–Oseretsky Test of Motor Proficiency, dental simulation, dental student, fine motor skill, preclinical dental education, skill acquisition

## Abstract

**Objective:**

The acquisition of motor skills is a key competency for the practice of dentistry, and innate abilities have been shown to influence motor performance. Thus, finding the most efficient manual dexterity tests may predict performance of dental students. The current study used the Bruininks–Oseretsky Test of Motor Proficiency, to assess motor skills of first year (D1) and second year (D2) dental students.

**Materials and methods:**

Three fine motor subsets of the BOT‐2—fine motor precision, fine motor integration, and manual dexterity—were administered to D1 and D2 dental students in 2017 and 2018. The BOT‐2 subset scores of D1 students were compared with those of D2 students, who had preclinical dental experiences. For D2 students, we tested for correlations between BOT‐2 subset scores and performance scores in a preclinical operative dentistry course.

**Results:**

No differences were found between D1 and D2 students for any BOT‐2 subtest scores (all *P*s > .09). No correlations were found between total scores of each BOT‐2 subtest and the operative dentistry course for D2 students (all *P*s > .20).

**Conclusions:**

Our results suggested the BOT‐2 was not predictive of manual skills of dental applicants or preclinical dental students. Although we assumed students would perform well with instruction, practice, and feedback, we were unable to determine whether innate abilities influenced acquisition of manual dexterity skills. More research about the acquisition of technical clinical skills in dentistry is required.

## 1 INTRODUCTION

The profession of dentistry requires identification of clinical competence through dexterity and other fine motor skills. Worldwide, admission to dental schools is often based on academic success, cognitive factors, and interpersonal characteristics. In the United States, dental schools have traditionally relied on applicants' predental cumulative and science grade point averages and on Dental Admission Test (DAT) scores (Ranney, Wilson, & Bennett, 2005). However, these factors have been shown to have limited predictive value of academic performance in dental school (Curtis, Lind, Plesh, & Finzen, 2007). Thus, the American Dental Education Association recommended the use of noncognitive methods in conjunction with traditional cognitive measures when dental schools make admissions decisions (American Dental Education Association, 2017).

Prior to 1972, a subtest called the Manual Average Score (average of the Spatial Relations Test and the Chalk Carving Test) was part of the DAT. The Chalk Carving Test, which is a test of manual dexterity, was the only noncognitive component of the DAT. However, it was controversial as a predictor of dental student performance (Chen, Podshadley, & Shrock, 1967; Fernandez‐Pabon, 1968), so it was replaced by the Perceptual‐motor Ability Test. Despite this change, there is a need to identify a screening tool for the DAT that more precisely predicts the performance of students in preclinical practical courses.

Dexterity research in the health professions has investigated a number of tests that mimic professional tasks (Gansky et al., 2004; Giuliani et al., 2007; Halstead, 1947; Kothe, Hissbach, & Hampe, 2014; Luck, Reitemeier, & Scheuch, 2000; Lundergan, Soderstrom, & Chambers, 2007; Wang et al., 2011; Wilson, Waldman, & MacDonald, 1991). For example, when completing the O'Connor Tweezer Dexterity Test (Lundergan et al., 2007), participants use tweezers to place pegs in a pegboard. The Hamburg Assessment Test for Medicine–Manual Dexterity (HAM‐Man), a wire‐bending test, is used for measurement of manual abilities in preclinical laboratory courses (Kothe et al., 2014). Even though a variety of tests have been assessed, there is no consensus on the best predictive test of manual dexterity (Suksudaj, Townsend, Kaidonis, Lekkas, & Winning, 2012). The Bruininks–Oseretsky Test of Motor Proficiency, Second Edition (BOT‐2), may be a possible screening tool for admission committees to assess the manual dexterity of prospective dental students. The BOT‐2 is a norm‐referenced standardized test developed by Bruininks (Bruininks, 1978; Bruininks & Bruininks, 2005; Deitz, Kartin, & Kopp, 2007) that assesses motor performance. More specifically, it measures fine manual control, manual coordination, body coordination, and strength and agility. Although it has not been validated as a predictive agent, it may be useful prior to enrolment as a predictor of performance. Therefore, it would be beneficial to determine if there is a correlation between BOT‐2 scores and performance in dental school.

The acquisition of motor skills is an essential competency for dental students, and research suggests that innate abilities may influence motor performance (Schwibbe, Kothe, Hampe, & Konradt, 2016; Suksudaj et al., 2012). The first year of the predoctoral dental curriculum includes courses in the basic sciences, dental anatomy, head and neck anatomy, and inflammation. These courses are meant to build on the students' existing knowledge of the sciences needed to provide oral health care. The second year curriculum emphasizes development of the skills needed for dental techniques and the fundamentals of the dental sciences. However, our understanding is incomplete regarding skill acquisition in dentistry and a student's innate ability to acquire those skills.

Therefore, the purpose of the current study was to assess the motor skills of first year (D1) and second year (D2) dental students using the BOT‐2. Specifically, we wanted to determine whether the BOT‐2 could be used as a noncognitive indicator of preclinical operative dentistry performance. We hypothesized that BOT‐2 performance would differ between D1 students, who had no preclinical experience, and D2 students, who had preclinical dental experiences with D2 students having higher performance. We also hypothesized that BOT‐2 scores of D2 students would be correlated with scores from a preclinical simulation operative dentistry course.

## 2 MATERIALS AND METHODS

### 2.1 Participants

All D1 and D2 students enrolled in a U.S. dental school during the 2017–2018 academic year were eligible for participation. Students were recruited to participate in the study by e‐mail invitation with multiple follow‐up e‐mails sent. All students held undergraduate degrees, and 12% of students in each class held advanced degrees. The study was reviewed by the local institutional review board and was considered exempt. The results of the BOT‐2 were confidential and were not shared with faculty. Each participant signed informed consent before participating in the study.

### 2.2 Procedure

Three of the four BOT‐2 fine motor subtests—fine motor precision, fine motor integration, and manual dexterity—were used in the current study to assess motor skills of dental students. The upper limb coordination fine motor subtest was excluded from the study because we did not believe it was relevant for assessing dexterity skills routinely performed by a dental student or practitioner. Three different examiners administered the BOT‐2 subtests, with each student being tested by only one of the examiners. Before data collection, the subtests were completed by seven university employees so examiners could practice standardization of test administration. All tests were completed in a quiet space with one examiner and one student. The total time for completion was recorded for each student. The demographic characteristics of sex and age for all students were recorded. The D1 students completed the BOT‐2 before the commencement of preclinical simulation courses in the curriculum, and D2 students completed the test after approximately 90 days of preclinical operative dentistry simulation.

The preclinical operative dentistry simulation course included daily hand skill projects, two objective structured clinical evaluations, one mock progress examination (Class II amalgam), and three progress examinations (Class II amalgam, Class II composite, and Class III/IV composite). The scoring system for assessments used a Likert scale (0–5) with half‐point increments. The minimum passing score was a 3.5. D2 students had 147 hr of preclinical time scheduled for the preclinical operative dentistry course.

The BOT‐2 fine motor precision subtest included seven different tasks. The tasks were filling in shapes (two tasks), lines through a path (two tasks), connecting dots, folding paper, and cutting paper. The fine motor integration subtest included eight tasks that consisting of drawing one or more objects. The manual dexterity subtest included five tasks that consisting of making dots in circles, transferring pennies, transferring pegs into a pegboard, sorting cards, and stringing beads. The BOT‐2 kits used in the current study were purchased from Pearson Education (New York, NY).

### 2.3 Statistical analysis

Descriptive statistics were used to summarize the demographic characteristics of participating students. Student performance was compared between D1 and D2 students for total scores and for each task within the three subtests of the BOT‐2 using the Wilcoxon rank sum test. Spearman's correlation coefficient was calculated for all tests within each subtest fine motor precision, fine motor integration, and manual dexterity subtests. For D2 students, we analyzed correlations between all test scores (i.e., total scores for each subtest and each task within the three subtests of the BOT‐2) and scores in the preclinical operative dentistry simulation course using the Spearman correlation coefficient. Two tasks within the subtests, drawing lines through paths‐crooked and copying a wavy line, had no variance in student scores, so correlations could not be calculated. The D2 students were subdivided into two groups based on their scores in the operative dentistry course: those who scored above average (high scorer) and those who scored at or below average (low scorer). Total scores of the three subtests of the BOT‐2 were compared between the high scorers and low scorers using a two‐sample *t* test. A *p* < .05 was considered significant. Data analyses were performed using SAS (Version 9.4, Cary, NC).

## 3 RESULTS

Eighty‐three students, 41/42 (98%) D1 students and 42/42 (100%) D2 students, participated in the current study. Of D1 students, 19 (46.3%) were females, and 22 (53.7%) were males; of D2 students, 17 (40%) were females, and 25 (60%) were males. The mean age for D1 students was 24 years (*SD* = 2.57), and the mean age for D2 students was 26.8 years (*SD* = 3.86). The mean total time to complete BOT‐2 fine motor subtest was 13 min (*SD* = 2.6) with a range of 8–20 min.

The mean final score for the preclinical operative dentistry course for D2 students was 87.71%. The maximum score for the course was 94.91%, and the minimum score was 78.59%.

The mean rank scores for total scores and for each task of the three subtests of the BOT‐2 are reported in Table 1. Mean total rank scores were 41.8 for D1 students and 42.2 for D2 students for the fine motor precision subtest, 42.3 for D1 students and 41.7 for D2 students for the fine motor integration subtest, and 41.7 for D1 students and 42.3 for D2 students for the manual dexterity subtest. No differences were found between D1 and D2 students for any BOT‐2 subtest scores (all *p* > .09).

**Table 1:** Comparisons between first year (D1) and second year (D2) dental students for the three fine motor subtests of the Bruininks–Oseretsky Test of Motor Proficiency, Second Edition

Subtest	Median (Q1–Q3)^a^		Mean rank scores^b^		p value
	D1	D2	D1	D2	
Fine motor precision	40 (39, 41)	40.5 (39, 41)	41.8	42.2	.94
Filling in shape‐circle	3 (3, 3)	3 (3, 3)	40.9	43.0	.48
Filling in shape‐star	3 (3, 3)	3 (3, 3)	43.0	41.0	.60
Drawing lines through paths‐crooked	7 (7, 7)	7 (7, 7)	41.5	42.5	.49
Drawing lines through paths‐curved	7 (6, 7)	7 (7, 7)	41.8	42.2	.92
Connecting dots	7 (7, 7)	7 (7, 7)	42.2	41.8	.93
Folding paper	7 (7, 7)	7 (7, 7)	39.9	44.1	.19
Cutting out a circle	7 (7, 7)	7 (7, 7)	43.1	41.0	.38
Fine motor integration	37 (36, 39)	37 (36, 38)	42.3	41.7	.91
Copying a circle	4 (3, 4)	4 (3, 4)	43.7	40.4	.50
Copying a square	5 (5, 5)	5 (5, 5)	38.8	45.1	.09
Copying overlapping circle	5 (5, 6)	5 (5, 6)	46.0	38.1	.11
Copying a wavy line	4 (4, 4)	4 (4, 4)	41.5	42.5	.49
Copying a triangle	5 (5, 5)	5 (5, 5)	39.9	44.1	.28
Copying a diamond	5 (5, 5)	5 (5, 5)	43.0	41.0	.68
Copying a star	4 (4, 5)	5 (4, 5)	39.8	44.1	.39
Copying overlapping pencils	5 (5, 5)	5 (5, 6)	41.6	42.4	.84
Manual dexterity	34 (32, 35)	34 (32, 36)	41.7	42.3	.90
Making dots in circles	9 (8, 9)	9 (8, 9)	40.8	43.1	.63
Transferring pennies	7 (7, 8)	8 (6, 8)	40.6	43.4	.58
Placing pegs into a pegboard	6 (5, 7)	6 (6, 7)	40.7	43.3	.62
Sorting cards	7 (7, 7)	7 (7, 7)	42.1	41.9	.97
Stringing blocks	5 (5, 5)	5 (4, 6)	42.2	41.8	.96

a Median, first quartile (Q1) and third quartile (Q3) of data are reported.

b Wilcoxon rank sum test is based on ranks, so mean rank scores are reported as well.

Spearman correlation coefficients for tests within fine motor precision subtest were significant only for filling in shape circle and filling in shape star (*ρ* = .4, *p* = .0003), between filling in shape circle and drawing lines through paths‐curved (*ρ* = .3, *p* = .003), filling in shape star and drawing lines through path crooked (*ρ* = .2, *p* = .03), filling in shape star and drawing lines through path curved (*ρ* = .5, *p* ≤ .0001), and drawing lines through path crooked and folding paper (*ρ* = .3, *p* = .02). Within fine motor integration subtest, correlation was significant for copying a circle and copying a square (*ρ* = .4, *p* = .0005), copying a circle and copying overlapping circle (*ρ* = .3, *p* = .002), copying a square and copying a wavy line (*ρ* = .2, *p* = .03), copying a square and copying a triangle (*ρ* = .3, *p* = .01), copying a square and copying a diamond (*ρ* = .2, *p* = .04), copying a square and copying overlapping pencils (*ρ* = .3, *p* = .002), copying a wavy line and copying a triangle (*ρ* = .2, *p* = .04), copying a wavy line and copying a diamond (*ρ* = .2, *p* = .03), and copying a triangle and copying a diamond (*ρ* = .3, *p* = .02). Within the manual dexterity subtest, only significant correlations were between making dots in circles and sorting cards (*ρ* = .4, *p* < .0001), transferring pennies and sorting cards (*ρ* = .4, *p* = .0005), placing pegs into a pegboard and sorting cards (*ρ* = .3, *p* = .01), and sorting cards and stringing blocks (*ρ* = .3, *p* = .01).

Spearman correlation coefficients between all test scores (i.e., total and each task within the three subtests of the BOT‐2) and scores in a preclinical operative dentistry simulation course for D2 students are reported in Table 2. The correlation coefficient for total scores was .19 for the fine motor precision subtest, −.07 for the fine motor integration subtest, and .20 for the manual dexterity subtest. No correlations were found between total scores of each BOT‐2 subtest and the operative dentistry course for D2 students (all *p*s > .20). For each task of the three subtests, only the transferring pennies skill in the manual dexterity subtest was correlated with the operative dentistry course (*ρ* = .35, *p* = .02).

**Table 2:** Correlations between scores of second year dental students on the three fine motor subtests of the Bruininks–Oseretsky Test of Motor Proficiency, Second Edition, and scores in a preclinical operative dentistry simulation course

Subtest	Spearman correlation coefficient	p value
Fine motor precision	.19	.22
Filling in shape‐circle	.21	.18
Filling in shape‐star	.25	.10
Drawing lines through paths‐crooked	NA	NA
Drawing lines through paths‐curved	.13	.41
Connecting dots	.18	.25
Folding paper	−.007	.97
Cutting out a circle	.18	.25
Fine motor integration	−.07	.67
Copying a circle	−.07	.64
Copying a circle	−.16	.31
Copying overlapping circle	.27	.08
Copying a wavy line	NA	NA
Copying a triangle	.04	.78
Copying a diamond	−.04	.79
Copying a star	−.18	.25
Copying overlapping pencils	−.009	.95
Manual dexterity	.2	.20
Making dots in circles	.27	.09
Transferring pennies	.35	.02
Placing pegs into a pegboard	.003	.99
Sorting cards	−.11	.49
Stringing blocks	−.05	.75

*Note.* NA values were obtained when no variation was found in students' scores, and so no correlation could be calculated.

Of D2 students, 22 (52.4%) were higher scorers, and 20 (47.6%) were low scorers. Mean scores for the total scores of the three subtests of the BOT‐2 for high scorers and low scorers in the operative dentistry course are reported in Table 3. No differences were found between scorers for any of the subtests (all *p*s > .29).

**Table 3:** Comparisons between total scores of three fine motor subtests of the Bruininks–Oseretsky Test of Motor Proficiency, Second Edition, and scores of second year dental students in a preclinical operative dentistry simulation course

Subtest	Mean (SD)		p value
	High scorers (n = 22)	Low scorers (n = 20)	
Fine motor precision	39.8 (1.6)	39.5 (2.0)	.56
Fine motor integration	36.7 (1.6)	37.0 (1.8)	.67
Manual dexterity	34.0 (3.6)	32.9 (3.0)	.29

*Note.* D2 students who scored average or above average on operative dentistry course are high scorers, and others are low scorers.

## 4 DISCUSSION

The current study assessed the motor skills of D1 and D2 dental students using three of the four BOT‐2 fine motor subtests to determine whether the BOT‐2 could be used as a noncognitive indicator of preclinical operative dentistry performance during the dental school admissions process. We found no statistically significant differences in any BOT‐2 subtest scores between D1 and D2 students. Because D2 students had some preclinical dental experience, we also analyzed for correlations between scores on the BOT‐2 subtests and scores in a preclinical simulation operative dentistry course. No statistically significant correlations were found between total scores of the BOT‐2 subtests and the operative dentistry course. Only one statistically significant correlation was found for the transferring pennies task in the manual dexterity subtest of the BOT‐2 and the operative dentistry course. Even when subdividing D2 students into high and low scorers in the operative dentistry course, there were no statistically significant differences between groups for total BOT‐2 subtest scores.

Our finding of no differences between D1 and D2 students was surprising. Because D2 students were exposed to preclinical manual dexterity skills through dental simulation in an operative dentistry course during the first year, we expected them to have significantly better scores on the BOT‐2 than the D1 students. The lack of significance could be attributed to two reasons. First, the comparison was made between two different groups of students, and it is possible that the D1 students had better or equal innate manual dexterity skills than the D2 students. The second reason could be that the BOT‐2 may not be a valid tool to measure hand skill levels in adults.

A limitation of the current study was the small sample size of both groups of students due to small overall class size in the school. A second limitation is the comparison of one class of students to another, which could be addressed in a future longitudinal study assessing the validity of the BOT‐2 tool to predict preclinical performance of dental students in a single cohort of participants with testing in D1 and D2 years.

## 5 CONCLUSION

Results of the current study suggested that the BOT‐2 was not beneficial for assessing the innate abilities of dental students regarding the acquisition manual dexterity skills either for dental school admission or for a simulated preclinical operative dentistry course. Additional research is necessary to find and validate standardized, noncognitive instruments that predict dental student performance during the admissions process.

## CONFLICT OF INTEREST

The authors have no conflicts of interest or financial interests to disclose.
